# India Unburdening the Pandemic: Jabs and Talks

**DOI:** 10.7759/cureus.40991

**Published:** 2023-06-26

**Authors:** Eilene Basu, Madhu P Raj, Vishwanath Krishnamurthy

**Affiliations:** 1 General Practice, Ramaiah Medical College, Bangalore, IND; 2 Internal Medicine, Ramaiah Medical College, Bangalore, IND

**Keywords:** covid-19 vaccination, healthcare workers, mental health, global healthcare system, covid-19

## Abstract

The impact of COVID-19 on the global healthcare system was detrimental, and India was not an exception. A crucial part of India’s fight during the pandemic was the nation’s astonishing vaccination delivery. From actively curbing the spread of COVID-19 and managing the affected to initiatives for the vaccination of our vast country, India faced numerous challenges in the healthcare delivery system during the pandemic. India’s compassionate initiative to supply COVID-19 vaccines across the globe was remarkable. With the rising caseload and increasing case fatality, healthcare workers (HCWs) worked tirelessly to fight the battle against COVID-19. This left gruesome effects on their mental health, leading to various mental health problems. To alleviate such concerns, the government and many renowned institutions in India put forth recommendations, services, and assistance to those suffering. In a nutshell, the healthcare system in India faced countless challenges during the COVID-19 pandemic, but the course of action taken to combat those challenges was truly extraordinary.

## Editorial

Vaccinating a nation

The development of a vaccine against the novel strain of coronavirus was a major milestone achieved by the global healthcare system in the mitigation of the pandemic. While the Pfizer mRNA vaccine was the first to be approved by the Food and Drug Administration (FDA) on December 31, 2020, the Oxford-AstraZeneca vaccine was approved on February 16, 2021. Over the course of the year 2021, there were varying perceptions on the indigenous vaccine, concerns about its efficacy, drug interactions, the safety of vaccine administration in patients with preexisting comorbidities, possible adverse effects, and complications. The same queries and concerns followed with the approval of other vaccines in India, such as Covaxin and Sputnik V. These debates were reflected in varying levels of vaccine acceptance among the public and, more so, the healthcare workers (HCWs).

Despite successful results on hamsters and rhesus macaques in phase I and II of clinical trials, the approval of vaccine usage in the national interest even before publishing the results of phase III of clinical trials raised serious concerns [1]. It would be imperative to say that the challenges faced by the healthcare delivery system in India were not only regarding the logistics but also sociocultural, such as religious and superstitious beliefs regarding the etiology of diseases. With general awareness, broadcasts, and regular public updates, India saw a vaccine acceptance rate of 84% [1].

The Ministry of Health and Family Welfare, Government of India (MoHFW-GoI) launched a nationwide campaign following the emergency usage approval (EUA) of the Oxford-AstraZeneca vaccine/Covishield and an indigenous vaccine BBV152 (trade name: Covaxin). Covaxin was a tremendously successful example of a public-private partnership (PPP) between the Indian Council of Medical Research (ICMR) and Bharat Biotech India Limited (BBIL) [1]. It is imperative to credit the early research and production of vaccines to the National Institute of Virology (NIV) for their tireless efforts, making India the fifth country worldwide to isolate pure strains of SARS-CoV-2 virus and developing indigenous reagents and testing kits for the same [1]. The vaccination schedule was approved as two doses of the vaccine at an interval of 4-12 weeks for Covishield and four weeks for Covaxin. This was subsequently topped up by a COVID-19 booster dose (CBD).

As per definition, a fully vaccinated person refers to any individual having received at least two doses of either of the COVID-19 vaccines if not the subsequent booster dose [2]. As of May 23, 2023, a cumulative 2,206,601,276 doses have been administered to the population of India with over 1.02 billion first doses and 950 million registered second doses [2]. Although the initial phase of the vaccination drive was targeted at all HCWs and COVID-19 frontline warriors, the later phases were expanded to the general population.

With an initiative named “Vaccine Maitri,” India extended its arms of support worldwide. Under this initiative, a total of 162 million doses of vaccine were distributed to 96 countries all across the globe. The major beneficiaries of this scheme were The Netherlands, Myanmar, Bangladesh, and Nigeria.

While the incidence of COVID-19 was not significantly reduced even after vaccination, there was a drastic fall in the number of severe COVID-19 cases, hospitalizations, and deaths due to COVID-19 [3]. In a study done by Watson et al. [3], an estimated 4.2 million deaths were successfully avoided due to vaccination in India alone. Despite the World Health Organization (WHO) declaring COVID-19 as not an emergency, the number of active cases stands at 6,591 as of May 24, 2023 [2].

Mind matters matter

The COVID-19 pandemic has had a vast effect on the mental health of HCWs worldwide, who experienced anxiety, depression, and hopelessness. The grueling challenges faced in the management of patients with COVID-19 led to burnout and deteriorating mental health. Devastatingly, many HCWs succumbed to suicidal thoughts and ideation, and attempts, which have been reported in the literature [4].

George et al. [5] conducted a study in an urban slum in India, where HCWs providing necessary healthcare reported feelings of stress and worry about being exposed to or contracting COVID-19. The study also depicted the issues faced by them in terms of living in a neighborhood after being infected themselves, which were viewed negatively by the neighbors [4,5].

The stigma surrounding HCWs spreading the infection after a hard day at work also led to inadequate social support once they were infected, and this had a straining effect on their mental health [5]. There is plenty of literature surrounding these issues in India and also across the globe. These adequately signify the impact of COVID-19 on the worsening mental health among HCWs and its burden.

To mitigate these issues, the MoHFW-GoI encouraged those in need to utilize the toll-free helpline number (08046110007) to express their feelings or for counseling. The authorities introduced various resources on their portal, such as coping mechanisms and videos of yoga/meditation techniques.

The GoI also launched the “Aarogya Setu” smartphone application [2]. This software program simplified the nation’s needs concerning safe practice guidelines, information regarding quarantine/containment zones, making online doctor’s/vaccination appointments, updates about COVID-19 positive/negative status, etc. Similarly, telemedicine services to promote mental health were extended by many institutions, notable ones including the National Institute of Mental Health and Neurosciences (NIMHANS), All India Institute of Medical Sciences (AIIMS), and Indian Psychiatric Society along with telepsychiatry facilities for patients who were in quarantine or isolation by other institutes. The Ministry of Home Affairs, GoI, presented standard operating procedures to help curb the spread of COVID-19 and national directives that included recommendations for the same, such as sanitation of workspace, regulations on public gatherings, permission for certain offices/industries to continue operations, and the strict lockdown implementation.

Conclusions

It is safe to say that the mental health of HCWs should be of ongoing importance. Not only the government but also the society should equally play an active role in mitigating these issues. The battle against COVID-19 is not easy, which is leaving tragic effects on HCWs. Despite the monumental challenges faced by the healthcare system in terms of manpower, money, material, and geographical terrain, the whopping two billion cumulative doses reflect the success of the vaccination drive in India. These collectively demonstrate that the healthcare system in India is unassailable.
